# A comparison of the clinical effectiveness and costs of mental health nurse supplementary prescribing and independent medical prescribing: a post-test control group study

**DOI:** 10.1186/1472-6963-10-4

**Published:** 2010-01-05

**Authors:** Ian J Norman, Samantha Coster, Paul McCrone, Andrew Sibley, Cate Whittlesea

**Affiliations:** 1 King's College London, Division of Health and Social Care Research, London SE1 8WA, UK; 2 King's College London, Centre for the Economics of Mental Health, Health Services Research Department, Institute of Psychiatry, Box P024, De Crespigny Park, London SE5 8AF, UK; 3 University of Southampton, School of Health Sciences, University Road, Southampton, SO17 1BJ, UK; 4 King's College London, Pharmaceutical Science Division, Department of Pharmacy, Franklin-Wilkins Building, 150 Stamford Street, London SE1 9NN, UK

## Abstract

**Background:**

Supplementary prescribing for mental health nurses was first introduced in the UK in 2003. Since then, a number of studies have reported stakeholders' perceptions of the success of the initiative. However, there has been little experimental research conducted into its effectiveness. This paper reports findings from the first known study to compare the cost and clinical impact of mental health nurse supplementary prescribing to independent medical prescribing.

**Methods:**

A post-test control group experimental design was used to compare the treatment costs, clinical outcomes and satisfaction of patients in receipt of mental health nurse supplementary prescribing with a matched group of patients in receipt of independent prescribing from consultant psychiatrists. The sample comprised 45 patients in receipt of mental health nurse supplementary prescribing for a minimum of six months and a matched group (by age, gender, diagnosis, and chronicity) of patients prescribed for by psychiatrists.

**Results:**

There were no significant differences between patients in the nurse supplementary prescribers' group and the independent prescribers' group in terms of medication adherence, health status, side effects, and satisfaction with overall care. Total costs per patient for service use were £803 higher for the nurse prescribers' group but this difference was not significant (95% confidence interval -£1341 to £3020).

**Conclusions:**

No significant differences were found between the health and social outcomes of patients in the mental health nurse supplementary prescribers' group, and those prescribed for by the independent medical prescribers. The cost appraisal also showed that there was no significant difference in the costs of the two types of prescribing, although the pattern of resources used differed between patients in the two prescriber groups. The results suggest that mental health nurse supplementary prescribers can deliver similar health benefits to patients as consultant psychiatrists without any significant difference in patients' service utilisation costs.

## Background

In 2001 suitably trained nurses were permitted to prescribe from an extended formulary that included 120 different medicines within the areas of minor injury and illness, palliative care, and health promotion [1]. However, it was not until 2003 that mental health nurses were permitted to prescribe through the introduction of supplementary prescribing [2]. Supplementary prescribing is described by the Department of Health (DH) [2] as "a voluntary prescribing partnership between an independent prescriber (a doctor or dentist) and a supplementary prescriber (a pharmacist, nurse or midwife) to implement an agreed patient-specific Clinical Management Plan, with the patient's agreement." In order to be registered as a supplementary prescriber, nurses attend a 26-day university-based training programme, followed by a period of supervised practice by an experienced doctor.

Since 2006, the extended Nurse Prescriber Formulary has been discontinued and qualified independent nurse prescribers can now prescribe all licensed medicines from the British National Formulary including some controlled drugs [3]. This latest development, was described in a British Medical Journal (BMJ) editorial as "one of the most far reaching extensions of prescribing by nurses and pharmacists anywhere in the world" [4]. The British Medical Association responded to this new policy of opening up the entire formulary to nurses with dismay, expressing concerns about safety, and emphasising the importance of diagnostic training to prescribing. Avery and Pringle [4] were more positive, noting that training in diagnosis for non medical health professionals is becoming more widespread. They pointed to findings from an influential study [5] which showed that independent nurse prescribers prescribed for relatively minor conditions and that medically trained assessors had found that generally, they prescribed appropriately.

However, the BMJ editorial also pointed out that the evidence base to support the current policy of non medical prescribing was weak, and that it was "worrying that the policy was launched by the Department of Health before waiting for further evidence to accumulate, including studies that it has only recently commissioned." This paper reports on findings from one of these Department of Health (England) (DH)) commissioned studies; a national evaluation of supplementary prescribing by mental health nurses.

Much of the early literature on mental health nurse supplementary prescribing in the UK consisted of expert opinion, sometimes with the benefit of the authors having visited mental health care settings in the USA, in which nurses have exercised prescriptive authority for over 20 years [6,7]. Within this anecdotal literature there have been debates on: whether mental health nurses have sufficient psychobiological knowledge to undertake prescribing [8]; whether the legal protection of nurses undertaking the role is adequate [9]; and whether nurse prescribing has been introduced primarily as a cost cutting measure [10], given the lower salaries of nurses compared to doctors. Although there have also been concerns regarding nurse prescribers' academic knowledge and clinical experience [4], a recent national survey by Courtenay et al. [11] suggested that the qualifications and experience of many nurse prescribers exceeds the training guidance stipulated by the Nursing and Midwifery Council [12].

Nurse prescribers have been widely surveyed regarding their experiences of their new role. On the whole, they have been found to have positive views about the initiative [13-16]. Although early surveys suggested that some nurses were anxious about obtaining sufficient knowledge and skills to carry out prescribing [17], practicing non medical prescribers report gaining greater confidence and knowledge of medicine management through prescribing, as well as greater job satisfaction and increased credibility with other professionals [16,18]. In addition, despite some of the negative commentary voiced by the medical profession [4], those studies which have sought the views of NHS doctors on nurse prescribing have found that they are broadly supportive of the initiative if the nurse prescribers are well qualified and experienced, and aware of their own limitations and of the context in which they are prescribing [15,18]. Studies within mental health settings which have examined psychiatrists' views of nurse prescribing also suggest that they are largely supportive of the nurse prescribing role [15,19,20], with consultant psychiatrists appearing to be less concerned about independent nurse prescribing than their junior staff [19]. This is encouraging as the relationship between the nurse supplementary prescriber and consultant psychiatrist is likely to be key to the success of non medical prescribing.

Perceived benefits of mental health nurse supplementary prescribing were that it would provide quicker and more efficient patient access to medication, would increase patient choice, and would make better use of nurses' skills and knowledge [21]. However, the effectiveness of supplementary prescribing has been difficult to ascertain. Recent case studies of nurse prescribers (both supplementary and independent) working in non mental health settings [22,23] suggest that staff perceive that prescribing has helped them to provide more streamlined packages of care, involve patients more in prescribing decisions, be more responsive to patient needs, and to work more holistically. Specifically Courtenay et al [23] reported that nurse prescribers in dermatology services felt able to spend longer in consultations discussing medicines, and were able to make prescribing decisions that were better tailored towards their patients' lives. There has been little comparable published research conducted within mental health. However, mental health patients' expectations of prescribing have been found to be positive, although tempered with uncertainty with regard to nurses' prescribing skills [24]. One study of patients' views of supplementary prescribing found that patients, on the whole, valued the nurses' holistic approach to medicine management [25].

Nurse prescribing has also been identified as a potential way of improving medication management for patients. Latter [26] suggests that prescribing medicines provides a new opportunity to affect the way patients take their medicines through maintaining good relationships and providing appropriate information on medicines. Certainly non compliance with medication in mental health care is a substantial problem which has been strongly linked to patient rehospitalisation and relapse [27] and non adherence rates for antipsychotics have been estimated to be as high as 74% [28]. There are to date no published studies which quantify the impact of mental health nurse prescribing on concordance. However, there is evidence gathered by Latter et al. [26] that nurse prescribers are generally practicing with due adherence to the main principles of concordance.

The aim of the present study was to compare the outcomes of patients prescribed for by a mental health nurse supplementary prescriber with those in receipt of independent medical prescribing. In view of the importance of taking medication appropriately, the primary outcome utilised in the study was self-reported adherence to medication. Secondary outcomes included clinical and social outcomes, side effects and satisfaction. In addition a cost consequences analysis was undertaken which compared the service costs of patients in both groups.

## Methods

This study adopted a post-test control group experimental design in five mental health NHS trusts. The mental health nurse prescribers' group comprised patients who had been in receipt of supplementary prescribing for at least six months, and the comparison group comprised a matched sample that had been in receipt of independent medical prescribing for at least six months. Sample size calculations showed that a sample of 60 patients in each group would be sufficient to show that the mean (adherence) score for patients managed by nurse prescribers was equivalent to the mean score of patients managed by independent medical prescribers within (plus or minus) 0.47 with 80% power at the 5% level of significance [29]. Due to practical difficulties involved in recruiting patients within tight time constraints and matching patients appropriately, the sample size was not achieved. The effect size was recalculated on the basis of the actual sample recruited which was 45 matched pairs (n = 90) and was found to be 0.54, with 80% power at the 5% level of significance.

Subjects were drawn from NHS trusts in which mental health nurse supplementary prescribers provided a service for a minimum of ten patients, with a primary diagnosis of depression, anxiety or schizophrenia, for at least six months. All patients were recruited to the study by a member of the research team. Each of the consenting patients in receipt of nurse supplementary prescribing were matched on primary diagnosis, gender, age group (within ten years) and length of time since diagnosis as a measure of chronicity (within ten years) to a patient whose medication had been managed by a consultant psychiatrist working in the same local area of practice. The only exclusion criterion applicable to patients from both groups was being detained under the Mental Health Act (1983) to ensure that consent was informed. In the event of more than one possible control being available for a patient in the supplementary prescribing group, a match from the independent medical prescriber's caseload was drawn at random. The study was approved by the Oxford REC A (Ref: 06/Q1604/39) and was completed in 2007.

### Outcome measures

Patients in the nurse prescriber and independent prescriber groups were interviewed using a structured interview (face to face or by telephone) comprising the following scales:

• The five item Medication Adherence Report Scale (MARS) [30] to measure medication adherence (primary outcome);

• The 19 item Satisfaction with Information about Medicines Scale (SIMS) [31] to measure satisfaction with medication information;

• The Beck Depression Inventory for patients with a primary diagnosis of depression [32] or the one item quick rating of depressed mood in patients with anxiety disorders [33] to measure depression in patients with anxiety and schizophrenia;

• The Work and Social Adjustment Scale (WSAS) [34] to assess social functioning and impairment;

• The one item Clinical Global Impression of Improvement scale (CGI) [35] to assess the patient's perception of improvement in their health problem;

• The Short Assessment of Adverse Effects of Anti-psychotic Medication Checklist [36]. The version used in this study was adapted from the original checklist to exclude those side effects which needed to be assessed or observed by a clinician;

• The eight item Client Satisfaction Questionnaire (CSQ-8) [37] to measure satisfaction with treatment from the main care provider/organisation;

• To examine patients' satisfaction specifically with their prescriber, the following question was included: "how satisfied are you with the person who prescribes your medicines?" Responses were rated on a four point scale from 1(very dissatisfied) to 4 (very satisfied).

### Costs

A cost consequences analysis was undertaken in which costs are reported alongside the multiple outcomes which were investigated. The analysis was undertaken from a health and social care perspective, which included informal care from family/friends. The following costs were included in the analysis:

#### 1. The cost of the intervention (mental health nurse supplementary prescribing)

This information was obtained from staff in the study sites and included training costs (time off work to complete the prescribing training course, continuing professional development, the cost of the supplementary training course including travel expenses, book allowances, and time spent in supervision) and the costs of the mental health nurse supplementary prescribing consultation (time spent preparing for a prescribing consultation, time taken to prescribe for a patient, and the number of patients prescribed for.) We attempted to include the costs of medication and tests ordered by the prescribers, but the information provided by staff was vague and so considered unreliable. It was therefore not included within the final calculation. In addition, although some data on consultation time with patients were recorded by nurses, consultation costs were finally calculated using data collected from patients as described below. The median cost per patient was used in the analyses, and sensitivity analyses were conducted whereby the number of patients treated was changed.

#### 2. Service costs of those patients prescribed to by nurses or by doctors

Service utilization costs from patients in the nurse and independent prescriber groups were collected using a shortened version of the Client Services Receipt Inventory (CSRI) [38]. The CSRI is one of the most commonly used measures of service costs. Its reliability and validity has been established and it has the additional advantage of enabling calculation of current standard costs for services [38]. Information collected by the CSRI included patients' use of health (primary and secondary) care, social care inputs, and informal (unpaid) care from family/friends as a result of the patients' health problems. Use of these services was measured retrospectively for the twelve months prior to interview. Service costs for the period were calculated by combining service use data with unit costs for 2005/6 [39]. These unit costs are derived by defining annual staff costs plus overheads (on-costs, training, capital, land, etc) and an appropriate unit of activity (e.g. hour of patient contact, inpatient day, etc). Informal care costs were estimated by combining the weekly hours of care with the unit cost of a homecare worker [39]. This was based on the assumption that in the absence of the carer it would be a homecare worker who would be needed to provide this type of help.

All analyses were performed using SPSS for Windows version 14. Paired t-tests were conducted to examine differences in the key outcome measures (Medical Adherence Report Scale, Satisfaction with Information about Medicines Scale, Work and Social Adjustment Scale, Beck Depression Inventory-II, One item Depression measure, the modified version of the Short Assessment of Adverse Effects of Anti-psychotic Medication Checklist, Clinical Global Impression of Improvement Scale, the unvalidated satisfaction with prescriber item and Client Satisfaction Questionnaire) between the two groups (i.e. the independent medical prescriber and the mental health nurse supplementary prescriber). Completed outcome scales which had one or more missing items were eliminated from the analysis. As this strategy resulted in very little data loss, it was not necessary to consider the use of pro-rating data techniques. We did not adjust for multiple testing as, with the exception of adherence, we were not testing specific hypotheses about the impact of either form of prescribing, and the issue of multiple testing is less crucial in exploratory analyses.

Cost differences for total service costs with and without informal care were tested for statistical significance using regression analysis with cost as the dependent variable and the group identifier as the independent variable. Cost data are usually skewed and therefore 95% confidence intervals were constructed using bootstrapping.

## Results

### Sample characteristics

Seventy two (87.8%) of the patients who were approached for recruitment to the mental health nurse supplementary prescribing group agreed to participate in an interview. Eleven of these patients were interviewed, but were subsequently dropped from the data set because suitable matches from psychiatrists' caseloads could not be found before the project ended, or their identified matches dropped out. Six patients declined to be interviewed after providing consent. A further eight patients were never interviewed because suitable matches from psychiatrists' caseloads could not be found before the study ended. Two were unavailable for interview after consent was taken due to re-hospitalisation. Fifty-five (79.7%) of the patients who were approached for recruitment from the independent medical prescribers' group consented to be interviewed; six were matched with patients in the nurse supplementary prescribers' group who were subsequently not interviewed, two declined to be interviewed after providing consent, and two were also unavailable for interview.

Demographic characteristics for the entire sample of 90 participants (45 matched pairs) are shown in Table 1. The mean age of the participants was 44.51 with a range of 23 to 71 years, and 54.4% (57) of the sample were men. Schizophrenia and depression were the largest diagnostic groups, with the remainder of patients diagnosed with either bipolar disorder, schizoid affective disorder or anxiety.

**Table 1:** Sample characteristics (n = 90)

Characteristics	Mean (SD)
Age (M) (SD)	44.51 (10.84)

Gender	54.4% Male 45.6% Female

Chronicity (years since diagnosis) Range: <1-15	8.69 years (3.18)

**Diagnosis**	

Depression	30 (33.3%)

Bipolar	24 (26.7%)

Schizophrenia	32 (35.6%)

Schizoid affective	2 (2.2%)

Anxiety	2 (2.2%)

The matching of subjects in the study was the closest that was possible on available demographic and health related criteria in an attempt to establish equivalence between the intervention and comparison group. There were no significant differences between the matched groups in terms of age or chronicity (Table 2). Ninety one percent of patients in the nurses' group (41/45) had been in receipt of supplementary prescribing for a period of 12 months or longer, which exceeded the minimum required prescribing period of six months.

**Table 2:** Differences between matched groups on matching characteristics

Sample	N	Nurse prescriber	Medical prescriber	
		**Mean (SD)**	**Mean (SD)**	**Sig (p)**

Age	45	44.10 (11.13)	45.11 (10.58)	0.732

Chronicity	45	9.16 (3.59)	8.20 (2.66)	0.101

### Clinical outcomes and social functioning

Table 3 compares the scores of the two groups of patients on the nine outcome measures. There were no significant differences on the primary outcome of self reported adherence to medication regimes, as assessed by the Medical Adherence Report Scale. There were also no significant differences found on any of the other validated scales (the Satisfaction with Information about Medicines Scale, the Work and Social Adjustment Scale, the Beck Depression Inventory-II, the One item Depression measure, the modified version of the Short Assessment of Adverse Effects of Anti-psychotic Medication Checklist, the Clinical Global Impression of Improvement Scale, and the Client Satisfaction Questionnaire). The comparatively large confidence interval calculated for the Beck Depression Inventory (BDI) measure, is a reflection of a greater number of patients in the independent prescribers' group with high BDI scores.

**Table 3:** Comparison of health and social outcomes between the independent prescriber and nurse supplementary prescriber groups.

		Nurse prescriber	Medical prescriber	95% CI of mean difference
	**N**	**Mean (SD)**	**Mean (SD)**	

SIMS overall score (0-17 : 17 = high satisfaction)	88	12.7 (4.22)	12.13 (4.33)	-1.45 to 2.40

MARS (5-25: 5 = high adherence	88	21.25 (4.09)	21.90 (2.68)	-2.25 to 0.94

WSAS: Work & Social Adjustment Scale (0-40 : 40 = severe)	89	17.18 (17.05)	16.72 (10.08)	-4.17 to 4.62

Clinical Global Impression Scale (1-7 : 7 = severe)	88	3.56 (1.47)	3.22 (1.49)	-0.24 to 0.91

BDI-II Mean Score (Depression/Bipolar only patients) (0-63 : 63 = severe depression)	46	17.95 (17.06)	20.69 (14.08)	-9.72 to 5.42

One item depression scale (Anxiety, Schizophrenia patients only) (1-8 : 8 = severe)	36	3.25 (1.98)	3.11 (0.83)	-0.67 to 1.04

CSQ-8 (8-32 : 32 = high satisfaction)	89	21.43 (5.35)	25.17(4.06)	-1.63 to 2.18

Side effects score (0-14; 14 = high prevalence of side effects)	90	4.06 (2.57)	4.44 (2.71)	-1.46 to 0.70

Satisfaction with prescriber (1-4 : 4 = high satisfaction)	90	3.42 (0.87)	3.04 (0.79)	0.03 to 0.72

There was a significant difference between the mean scores of the two groups on the satisfaction item. Patients in the nurse prescriber group reported a higher level of satisfaction with their current prescriber than those patients in the independent medical prescriber group

### Economic outcomes

Table 4 summarises information on service use and costs. Notably patients in the independent medical prescriber group had a greater mean frequency of contacts with other therapists (e.g. art therapist, specialist chronic fatigue therapist) than those in the nurse prescriber group (10.1 mean contacts versus 35.0 mean contacts) and a similar pattern emerged for psychiatric day care (14.5 mean contacts versus 56.0 mean contacts).

**Table 4:** Service use and costs per patient over 12 months of mental health nurse supplementary prescribing compared to independent medical prescribing

	Nurse supplementary prescriber group			Independent medical prescriber group		
**Service**	**N (%) using Service**	**Mean (SD) Contacts**^1^	**Mean (SD)** **Cost (£)^2^**	**N (%) using service**	**Mean (SD)** **Contacts^1^**	**Mean (SD** **Cost (£)**^2^

Intervention	45 (100)	-	497 (0)	0 (0)	-	0 (0)

Medical outpatient/Daycare	9 (20)	5.1 (5.1)	99 (297)	4 (9)	3.8 (3.8)	33 (143)

Psychiatric day care	10 (22)	14.5 (27.1)	76 (304)	8 (18)	56.0 (51.8)	312 (1154)

Non-psychiatric nurse	21 (46)	6.6 (8.3)	89 (287)	12 (27)	7.5 (13.3)	113 (612)

Other therapist	8 (17)	10.1 (11.4)	79 (324)	6 (14)	35.0 (40.3)	340 (1274)

Psychiatrist	33 (72)	8.9 (9.6)	612 (1012)	37 (84)	13.9 (9.2)	1032 (972)

OT/vocational worker	4 (9)	2.8 (2.4)	6 (25)	1 (2)	5.0 (-)	5 (30)

Social care	6 (13)	11.6 (8.8)	106 (379)	8 (18)	18.1 (8.3)	181 (613)

Psychiatric inpatient^3^	9 (20)	52.1 (44.1)	2049 (5622)	4 (9)	47.3 (49.8)	863 (3824)

Medical inpatient^3^	5 (11)	3.0 (0.7)	79 (235)	1 (2)	7.0 (-)	39 (256)

GP	33 (72)	8.3 (11.4)	159 (314)	40 (91)	5.0 (4.2)	140 (163)

Psychologist	3 (7)	13.8 (1.1)	59 (227)	3 (7)	15.0 (8.9)	68 (282)

CMHN	35 (76)	22.1 (16.5)	603 (600)	16 (36)	36.4 (20.7)	594 (1008)

A&E	6 (13)	1.2 (0.4)	13 (37)	2 (5)	1.5 (0.7)	6 (29)

Informal care	17 (37)	1196 (994)	6188 (11645)	16 (36)	1037 (1368)	5278 (13336)

**Total excluding informal care**			**4526 (5996)**			**3723 (4391)** **(95% CI, £-1341 to £3020)**

**Total including informal care**			**10714 (13257)**			**9001 (14465)** **(95% CI, £-3950 to £6699)**

^1 ^Contacts for those using services only

^2 ^Costs in 2005/6 £s and for whole sample

^3 ^Contacts = number of days

Mean psychiatric inpatient user costs per patient were substantially higher for the nurse prescriber group than those in the medical prescriber group (£2049 versus £863). This reflects the fact that twice as many patients in the nurse prescriber group (n = 9, 20% of the total nurse prescriber sample) had an inpatient stay during the previous 12 months compared to those patients in the independent medical prescriber group (n = 4, 9% of the total medical prescribers' sample). Although this amounted to only five more patients in the nurse prescriber group who experienced psychiatric inpatient stays, the costs of these were so great compared to all other costs that they led to a substantial difference in costs between the two forms of prescribing.

The total annual cost per patient, excluding informal care, was £803 higher for the mental health nurse supplementary prescribers' patients, but this difference was not significant (95% CI, -£1341 to £3020). The costs including informal care were £1713 higher for the nurse prescriber group but again this was not significantly different (95% CI, -£3950 to £6699).

**Figure 1:**
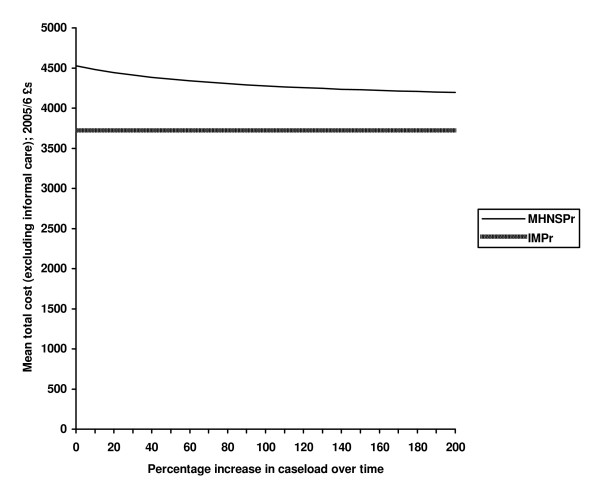
**Sensitivity analysis: service costs for nurse and independent prescribers' groups as nurse prescriber caseload increases**.

Figure 1 shows the impact on total service costs (excluding informal care) as the caseload (or number of patients prescribed for by the nurse) increases as a function of time. The effect of the increased number of patients reduces the unit cost of the intervention, but the figure shows that this has a modest effect on total costs. Even if numbers increased by 200% the costs would remain above those for the independent medical prescriber group, although the difference would not be significant.

### Cost of implementing supplementary nurse prescribing

An additional cost to be considered by trusts which decide to establish a nurse supplementary prescribing service is the cost of nurse prescriber training, which we calculated per patient at £497. Other costs would include those for professional updating and supervision by doctors. Although such data were collected during the course of the study, no attempt to calculate an average for these additional costs was made.

## Discussion

The study demonstrates that there were no significant differences in self reported adherence to medication between patients who had been prescribed for by a nurse prescriber for a period of at least six months and those prescribed for by an independent medical prescriber. Patients in the nurse supplementary prescriber group did report significantly higher satisfaction with their prescriber on an unvalidated measure, but as this was an exploratory analysis with improvement in satisfaction not being an *a priori *hypothesis, and given the number of tests conducted, it may have been a chance finding. There was no difference found on the validated satisfaction measure, the Client Satisfaction Questionnaire. However as this scale was developed to assess satisfaction with all aspects of healthcare, it is therefore likely that service users considered the care received from a range of health professionals in addition to their prescriber when rating this scale. Further research is needed to develop self-report measures which can reliably quantify mental health patients' satisfaction with supplementary prescribing.

The economic evaluation of mental health nurse supplementary prescribing is particularly important given the finding of no significant difference between adherence, and the clinical and social outcomes of patients in receipt of supplementary prescribing and independent medical prescribing. If both forms of prescribing are equivalent, then a key question is which one is less expensive? Our cost appraisal shows no significant difference in the costs of the two types of prescribing. However, the pattern of resources used differed between patients in the two prescriber groups. Service users in the independent medical prescribers' group had a greater mean contact with other therapists (e.g. art therapist, specialist chronic fatigue therapist) and reported more use of psychiatric day care than those in the nurses' group although differences in these costs were not significant. Patients in the nurse prescribers' group were more likely to have been admitted as psychiatric inpatients during the previous 12 months, than those patients in the independent medical prescribers' group. However, it is not possible to ascertain the reason for this difference in admission days. Given that there were only five more patients (out of a total sample of 90) requiring inpatient treatment in the mental health nurses' group, this might be a chance finding. Or it might be that nurses are more inclined than doctors to refer patients whose behaviour gives rise to concern to inpatient care facilities; this may reflect their lesser experience and possibly their lower level of confidence in their skills of risk assessment than doctors, whose patients appeared to utilise day services more often.

The finding that the nurses' group had more inpatient stays is particularly interesting, given the fact that the independent prescriber group had a higher depression score (although not significantly) than the mental health nurses' group. The independent prescribers' group may have been more severely ill, because more severe or unstable cases might have been retained by the doctors, whilst the more chronic and stable cases might have been transferred to mental health nurses. However, this is speculation as we have no firm evidence from this study that illness severity was a criterion which determined case allocation to the nurses.

### Strengths and limitations

This is the only known national evaluation of mental health nurse supplementary prescribing in the UK, and the first to adopt an experimental research design to compare outcomes and costs for patients managed by nurse supplementary prescribing and independent medical prescribing. Thus, the study represents an advance on expert opinion papers, and adds to previous research on the views of staff [13,14] and patients [24,25] towards nurse prescribing as a policy initiative.

The results of this study are encouraging for the future of supplementary prescribing since it suggests that prescribing by mental health nurses may be as effective as the 'gold standard', that is prescribing by independent medical prescribers. However, it would be premature to draw any overall conclusions on the basis of this single study. Whilst subjects in both groups were matched on several characteristics, it was not possible to match them at baseline (on commencement of nurse supplementary prescribing) on diagnostic specific, generic health or social care outcomes. This raises the possibility that one or other group of subjects was more seriously ill at baseline and so made more progress than the other over the prescribing period. Even if health outcome data had been available at baseline, drawing conclusions about causality would remain contentious in the absence of randomisation of subjects to the care of either the mental health nurse or the independent medical prescriber.

Patients' health outcomes in this study were assessed using self-report measures. Assessing adherence using clinicians' reports, pill counts or biological measures, are time consuming and are not always accurate [40]. Patient self-report methods also have disadvantages, and it has been suggested that self-report measures overestimate adherence rates [41]. However, given that no method is ideal, validated self-report scales are generally considered to be a cost-effective and time-efficient way of assessing adherence [42]. In addition, any over estimation of adherence by patients was equally likely to occur in both the nurses' and psychiatrists' groups, and so was unlikely to confound the results of this study.

In this evaluation we sought to calculate incremental costs, that is, the additional costs that would be incurred by NHS trusts on implementation of mental health nurse supplementary prescribing. It was beyond the scope of the evaluation to consider issues such as the economic effects of longer term changes in skill mix within trusts, which might arise if mental health nurse supplementary prescribing was adopted widely. Investing in professional skills is like any other investment, in that the returns come as a flow of benefits over time. In addition skills require maintenance and continuing development, and much will depend also on how long the nurse prescribers, once trained, continue to deliver a prescribing service for patients.

## Conclusions

Whether supplementary prescribing or independent prescribing is a desirable development must depend ultimately on its benefit for patients and, more specifically, whether it is as cost effective as the current standard system of prescribing by independent medical practitioners. The most important consideration when planning the future of mental health nurse supplementary prescribing is the experience and wellbeing of patients. This evaluation suggests that the effect on patients of transferring from an independent medical prescriber to a mental health nurse prescriber may be negligible, which is encouraging for the future of mental health nurse supplementary prescribing.

## Competing interests

The authors declare that they have no competing interests.

## Authors' contributions

IJN and CW were involved in the conception and design of the study, and obtained funding. SC, AS, PMC were responsible for the collection and analysis of study data. IJN drafted the manuscript with critical revision from SC, PMC, AS and CW. All authors have read and approved the final manuscript.

## Funding

The Department of Health (England). The paper represents the views of the authors and not necessarily those of the Department of Health. The study was commissioned by the funders and completed independently by the researchers.

## Pre-publication history

The pre-publication history for this paper can be accessed here:

http://www.biomedcentral.com/1472-6963/10/4/prepub
